# Recommendations for outpatient activity in COVID-19 pandemic

**DOI:** 10.1515/med-2021-0378

**Published:** 2021-11-09

**Authors:** Piergaspare Palumbo, Fanny Massimi, Antonio Biondi, Roberto Cirocchi, Giuseppe Massimiliano De Luca, Giorgio Giraudo, Sergio Giuseppe Intini, Roberta Monzani, Giampaolo Sozio, Sofia Usai, The Società Italiana di Chirurgia Ambulatoriale e Day Surgery (SICADS) Board

**Affiliations:** Department of Surgical Sciences, “Sapienza” University of Rome, Viale Regina Elena 324, 00161 Rome, Italy; Department of General Surgery and Surgical Specialities, University of Catania, Catania, Italy; Deparment of Surgical and Biomedical Sciences, University of Perugia, Perugia, Italy; Department of Biomedical Sciences and Human Oncology, University of Bari, Bari, Italy; Department of Surgery, Santa Croce e Carle Hospital, Cuneo, Italy; Department of General Surgery, University of Udine, Udine, Italy; Department of Anesthesia and Intensive Care, Humanitas Research Hospital, Rozzano (MI), Italy; Department of General Surgery and Emergency, Alta Val D’Elsa Hospital, Poggibonsi (SI), Italy

**Keywords:** day surgery, day case, COVID

## Abstract

The spread of the COVID-19 disease substantially influenced the International Healthcare system, and the national governments worldwide had before long to decide how to manage the available resources, giving priority to the treatment of the COVID-infected patients. Then, in many countries, it was decided to limit the elective procedures to surgical oncology and emergency procedures. In fact, most of the routine, middle-low complexity surgical interventions were reduced, and the day surgery (DS) activities were almost totally interrupted. As a result of this approach, the waiting list of these patients has significantly increased. In the current phase, with a significant decrease in the incidence of COVID-19 cases, the surgical daily activity can be safely and effectively restarted. Adjustments are mandatory to resume the DS activity. The whole separation of pathways with respect to the long-stay and emergency surgery, an accurate preoperative protocol of patient management, with a proper selection and screening of all-day cases, careful scheduling of surgical organization in the operating room, and planning of the postoperative pathway are the goals for a feasible, safe, and effective resumption of DS activity.

## 1 Introduction

COVID-19 pandemic is incontestably one of the greatest events with social and public health impact ever recorded in the history of humanity. Up to now (April 2021), over 141 million confirmed cases of infection and more than 3 million deaths are reported worldwide [1]. The Italian and European panorama is recording a considerable reduction of the infection curve of data, with an Rt index lower than 1 in all regions. The state of alert, however, remains very high. It is an unmatched medical emergency, being able to induce a sudden change in management of available healthcare resources for elective surgery, to reallocate funding to the COVID-19 patient’s care. The negative impact on surgery activities has been very significant. It is estimated that since February 2020 more than 30 million surgical procedures were canceled. During this emergency, the not strictly required surgical procedures, outpatient activities, and overall surgical benefits have been postponed. Only the emergency surgical procedures never suspended their activities. The oncologic surgery too, after an initial braking, quickly adjusted to new assistance pathways [2]. Instead, the day surgery (DS) recorded a complete cessation of intervention, and the dedicated operating rooms are no longer used. For this reason, these surgical models need to get valid, standardized recommendations to restart as quickly as possible [3].

Furthermore, in 2018 in Italy, more than a million of surgical procedures have been performed in a DS setting. This really means that, during the period from February 2020 to February 2021, more than a million procedures have been delayed. This sets a great problem in terms of patients retrieving once the activities will start again. Such conditions should not accept a further delay of the resumption of DS, especially observing a strong reduction in infection rate.

In our country, we can consider that, now, after a decrease in the incidence of COVID-19 cases, elective surgical activity has been prudently resumed. Indeed, according to the American College of Surgeons, the time of resumption for elective surgery is at least 14 days after the reduction of the cases [4]. In Italy, the sanitary authorities decided to adopt a more restrictive behavior for DS patients, and up to now, the outpatient wards and activities are only partially resumed. The aim of this article is to recommend a quick and safe resumption of work in DS units.

DS is commonly performed in free-standing units or dedicated hospital areas, with separated access to spaces for admission, and operating rooms exclusively reserved for daycare activities, with separate pathways regarding the emergency and long-stay units.

This allows safer and more appropriate activities and processes management. In this phase, only surgical facilities able to guarantee a true separation of pathways should be authorized to restart the activities.

In this phase, a limited but consistent number of patients should be considered asymptomatic or pre-symptomatic. Some precautions therefore must be adopted in the pre-, intra-, and postoperative processes.

Patients selected in DS might be COVID-19 negative, undergoing only an elective, nonurgent pathology generally treated in this setting. COVID-positive patients could be postponed to a more appropriate stage when the disease will be healed [5,6].

In different cases, if the disease does not allow a delay of care, patients must be sent to a common COVID pathway, as well as emergency and long-stay patients, not able to set back the surgical interventions.

Currently, the common behavior is to consider elective, nonurgent surgery on COVID-19-positive patients to be postponed.

In fact, the COVID-19-positive patients undergoing surgery have higher mortality than other patients with similar conditions in a non-COVID time [7].

According to Lei et al., who examined a series of 34 patients operated electively, surgery worsened the course of COVID-19 disease [8].

However, there is no reason to delay any longer the elective daily surgery on COVID-19-negative patients in the countries where the epidemic peak has been reached and the healthcare quality can ensure safe and ethically acceptable delivery of care.

In fact, morbidity and mortality following outpatient surgery are exceptional. In an experience conducted between 2014 and 2018 at the Kingston General Hospital (Ontario, Canada), only 2.9% of cases were rehospitalized after outpatient procedures, most of them for clinical observation [9], and emergency postoperative surgical procedures are extremely uncommon.

This article proposes an array of recommendations regarding surgical risk stratification and management of surgical procedures in a DS setting, at the COVID time. The implementation of protocols and recommendations can allow an early restart of DS procedures, remaining ready to stay active also in the unfortunate event of a possible further wave of the epidemic.

## 2 Risk management

*The rational approach to the risk management in DS must be based on the peculiarities of this surgical activity, typically characterized by high-flow production processes and with a high possibility of standardization*.

A proactive approach can be realized through a careful engineering process of clinical pathways. It is used by a variety of organizations, including the World Health Organization and the United States Federal Emergency Management Agency [10]. Process analysis is therefore essential in managing clinical risk; for each activity, an attempt is made to identify the errors that may occur during execution and the associated risk is quantitatively assessed. Once all possible adverse events have been identified, the probabilities with which they may occur must be assessed and therefore different priority requirements must be assigned to the various occurrences.

During the COVID-19 pandemic, DS allows to offer the patients a safe care setting, able to provide high-quality proposals, and personalization services in a short period of time. This is particularly important in the COVID-19 pandemic when the patient’s exposure time within a healthcare facility must be minimized with good effectiveness. The management of the daycare pathway must be directed to minimize the risk of in-hospital infection. Critical issues are as follows:

– The preoperative undiagnosed infection with evidence of the disease during hospital stay or after discharge.
– The infection transmission to or from the health staff.
– The need of an adequate pathway for the alert in case of contact with positive subjects.

In the high-income countries, the high number of elderly patients with comorbidity increases the risk of adverse events related to infection brought by silent healthy COVID-19 carriers [11].

## 3 Preoperative pathway

*The selection of the surgical procedures, allowing a fast recovery (no longer than 4 h) and safe discharge the same day of surgery, is mandatory*.

Patients selected for DS procedures in the descending curve of the COVD-19 circulation should be considered potentially positive, and appropriate precautions should be observed, without forgetting to implement the consent form with a sentence that explains to patients that is always possible to contract a COVID infection in the hospital, despite a safe therapeutic way is ensured [10,11].

A delicate issue concerns the acceptance of frailty patients: children and adults, who need to be assisted by a caregiver throughout their hospitalization. Only one career is admitted and must be submitted to the same precautions required to patients, as in the following description.

The main criteria for admission of patients in a DS unit will remain similar with respect to the pre-COVID time.

Primarily, the selection criteria universally acknowledged are still valid. We need to be careful during the evaluation of patients who have had COVID-19 who undergo surgery because they are at an increased risk of postoperative death. Postoperative pulmonary complications occur in half of the patients with perioperative SARS-CoV-2 infection and are associated with high mortality [11].

In the last years, a growing interest in terms of prehabilitation before surgical procedures is reported in the literature. Prehabilitation is considered as a process of optimizing physical functionality preoperatively to permit maintaining a normal level of function during and after surgery [12]. Prehabilitation could be useful in ambulatory surgery, especially during a pandemic: high-risk patients in the ambulatory setting should be identified, stratified, and optimized [13].

Moreover, the possibility to get a faster discharge, related to the government legal provisions about the people’s mobility, must be considered [14,15]. Nevertheless, the DS unit should be independently organized in terms of management of the preoperative pathway. When it would not be disconnected from other structures of the hospital, an independent space should be created, and the hospital structures adapted.

The exams and the anesthetic assessment should be undertaken in the same ward, without moving the patient. The routine blood exams should be supplemented, if possible, by the vitamin C and D level assay [16,17]. Chest X-ray, instead, unless necessary due to the patients’ clinic conditions, should be avoided.

Moreover, the possibility to get a faster discharge is related to the government legal provisions about the people’s mobility.

Patients should be carefully selected. COVID exposure, age, American Society of Anesthesiologists (ASA) score, and overall risk factors must be considered. At this stage, only patients with 1-2 ASA scores should be admitted, due to the increased risk of infection in people with comorbidity. The selection, in summary, should consider the clinical risk factors, and only intermediate or minor risk factors can be admitted [18,19].

Compared to the time before the COVID pandemic, closer attention is dedicated to the elderly. Patients with cerebral, cardiovascular, bronchopulmonary disease, renal failure, blood disorders, and diabetes should be carefully considered [20,21]. When a delay is not feasible, an adequate preoperative balance of comorbidities is mandatory. The agedness is currently considered not a risk factor in elderly patients with good performance status, but in this condition should be restrictively considered, due to the increased severity of the disease manifestation in the elderly population [22]. Obesity as well should be considered a risk factor [23]. We are faced, unfortunately, with a new virus of which we do not yet know all the characteristics, so the age of patients and comorbidities are the most important indicators of the intraoperative risk.

Very careful management is required to minimize the contact among patients themselves and with the health workers, particularly nurses: the physical separation signaling for 1.5 m distance should be indicated. The different pathways (acceptance area, admission room) should be highlighted by color dots on the floor.

Hand sanitizer dispensers must be distributed at each DS entrance. The workers and the patients must wear the PPE mask. Each patient can be accompanied by a person who waits outside and comes in only when the patient can be discharged. Preoperative consultations by the surgeon and anesthesiologists should be scheduled (30 min a patient), and it is important to respect the social distance in the waiting room.

Before accessing the operating room, a safety kit – mask, disposable gowns, gloves, must be prepared at each stay bed [24].

## 4 Screening

*A careful information of patients and accurate covid tests are essential. The RT-PCR is the most sensitive test to ensure a safe surgical procedure*.

The preoperative screening can start by phone or video calling, submitting some questions about their COVID conditions, as exposure, symptoms, disease development, and previous testing. All patients who are not known to have had COVID-19 and who are scheduled for surgery should be screened for exposure to COVID-19, and for symptoms (i.e. fever, cough, shortness of breath, muscle pain, sore throat, and/or new loss of taste or smell) within the prior 2 weeks; patients with symptoms should be referred for further evaluation.

Then, the patient must be carefully informed about the emergency that has reshaped the health system, and the safety systems that the hospital is able to ensure [25]. With the aim to assess the infectious condition, a preoperative COVID-19 test is essential. The reverse-rranscriptase polimerase chain reaction (RT-PCR) test is sensitive and reliable, easy to run, and provides results in a very short time [26].

In a Cochrane study [27], the RT-PCR test had an average sensitivity of 95.2% whereas the antigen test had a sensitivity of 56.2%. The antigen test correctly detects SARS CoV-2 in the presence of a high viral load, and its sensitivity rapidly declines when the viral load decreases [28].

As even asymptomatic patients and patients with negative results could be contagious, we should consider every patient who comes to our departments as potentially COVID-19 positive bringing us to implement safety measures and choosing pre-, intra-, and postoperative procedures that can expose less hospital staff and the other patients to the infection.

Considering that patients presumed to be infected must be excluded from DS, a systematic lung CT scan is unnecessary [29,30,31].

The RT-PCR test should be carried out 48–72 h before surgery, and, afterward, patients should self-isolate themselves up to the time of admission, even if patients are vaccinated. A trust agreement, in this sense, is mandatory [5].

There is no evidence that a rapid antigen test performed right before admission is demonstrated to be useful [32].

## 5 Surge protection device (SPD) usage

*A rational usage of SPD is central in epidemic phase because it ensures health worker protection and prevents disease diffusion.*

An operating room is an environment in which forced air circulation promotes dispersion of particles eventually contaminated. According to current thought, greater droplets remain next to the source, while smaller one is dispersed in major areas, being contamination aerosol source. Recent studies demonstrate that both, smaller and greater droplets, persist at a short distance from the diffusion source, which means that aerosol contamination happens also next to the source [33]. Proper usage of SPD is essential to prevent infective disease diffusion among patients and health care workers. However, due to the rapid diffusion of the pandemic, healthcare workers are not enough educated on the corrected usage of SPD. There are several studies built up on the importance of donning and doffing to prevent self-contamination at the end of procedures. Fluorescent markers were used to perform simulation, identifying at the end of procedures if there were personal self-contamination. Results demonstrate that healthcare workers diverge from protocol mainly in doffing; in fact, fluorescent marker remnants were found on the hands of the workers as detection of ineffective and high-risk procedures [34].

A study proposes active training through the exercise of donning and doffing or passive training viewing tutorials [35] and by the usage of a checklist with all steps for correct procedures. It is clear the need to realize studies with the goals of continuous education of health workers, not only in proximity to a pandemic but also to ensure correct usage of SPD and consequently a low risk of contamination.

## 6 Scheduling of surgery in the operating room

*Two groups of surgery candidates can be considered, with different pathways.*

(1) A group undergoing procedures not producing airborne droplets.
  These patients are submitted to surgery with local anesthesia, with continued monitoring, and locoregional anesthesia, including the central block. Moderate sedation or analgosedation is admitted. Many surgical procedures can be performed in this manner (hernia repair, minor urological procedures, varicose vein surgery, arthroscopy, and many others). Only upper gastrointestinal procedures should be excluded from this group [36].
  To reduce viral contamination during the surgical procedure, a dose of nasal povidone-iodine before the placement in the operating room can be useful [37]. Alcoholic sanitizers must be used by health professionals before and after any contact with patients [38]. Double gloves are used when the patient’s mouth or nose are touched to remove the external ones when the procedure is completed. Carefully wipe any surfaces that have been exposed and possibly infected by the patients. If oxygen needs to be administered, a nasal cannula under the surgical mask should be applied without using high flow [39,40].
  The number of surgeons, nurses, and other workers inside the operating room should be minimized. It is preferable to define one rotation of the residents/students if you work in a research/university hospital. Only then, it is possible to reduce the virus transmission and infection, involving not only the patients but also all the health professionals and workers.
  The operating time should be reduced to the strict minimum. Surgeons, nurses, and other workers need to use only disposable devices, surgical masks (FFP2 are the safest) and/or FFP2, high-quality sterile surgical gowns, and protective glasses (during nebulizer operations). All the instruments should be sealed in a sharp container, and those not disposable are transferred to the sterilizing room. After surgery, patients are quickly brought back to the ward, without staying in a recovery room. The necessary environment cleaning extends the time between each surgical procedure and could determine the performance of fewer surgical procedures for each day [14,15]. This problem could be resolved if the plan of work is distributed on 6 days/week and not only 5!
(2) A second group interested by procedures producing airborne droplets.
  In this group, upper gastrointestinal endoscopy, bronchoscopy, upper respiratory surgery, ORL surgery, maxillofacial surgery, and all procedures should be done under general anesthesia. These procedures require extreme cautions and specific procedures of environmental treatment, especially the use of double gloves during intubation and the maintenance of anesthesia. Special care is taken to the safe disposal of laryngoscope blades, or it is better if it is possible to use a video laryngoscope. The rapid sequence intubation (RSI) is strongly recommended [21,41], to avoid aerosolization during manual ventilation with a mixture enriched of oxygen; if manual ventilation is necessary, it is suggested to use little tidal volume and a close circuit for airway aspiration must be prepared. Total intravenous anesthesia could be preferred [42], but when it is possible it is suggested to choose locoregional anesthesia, with the purpose to consent to a rapid recovery. When general anesthesia is performed, a double filtration system, with high efficiency, must be allocated between the expiratory circuit, the ventilator, and the extremity of the breathing circuit connecting the patient to the ventilator [42].

The procedure’s turnover is significantly prolonged, considering that the presence of workers in the operating room is reduced, and the total number of treated patients per day is lower.

These procedures, whenever possible, should be delayed in this acute phase of COVID-19, or shifted to the general surgery operating rooms, considering specific COVID safety precautions. Accordingly, the surgical pathway is different than the one dedicated to outstanding patients.

If the local/regional anesthesia must be converted to the general one, all described precautions must be adopted.

However, local or regional anesthesia should be preferred, associated with sedation whenever needed. Unlike in the non-COVID period, general anesthesia should be preferably performed using a laryngeal mask, because the supraglottic devices do not ensure a complete seal of the airway, and a possible droplets contamination on the surgical room can occur [41].

## 7 Postoperative care

*During the postoperative phase, a close attention should be paid to respect social distancing between patients and use protection devices.*

After surgery, all patients, treated with any type of anesthesia, should be directly admitted to the ward for short monitoring (Levels 1–2) to reach an adequate Post Anesthesia Discharge Scoring System (PADSS) and discharged as soon as possible. In the case of general anesthesia, it is mandatory to awake and stabilize the patients in the theater. They should not stay in the recovery room, and a quick return to the ward is recommendable.

Reduced postoperative access in the hospital and fast recovery (no longer than 4 h) are recommended. Caregivers cannot be admitted since the patients are ready to discharge. The use of protection devices inside the ward, for patients and health professionals, is mandatory. Prescriptions and instructions should be already available at the time of discharge, so patients can be turned into caregivers without further transit to the reception [24]. The admission and discharge pathways are in this case completely separate. The use of the PADSS is highly recommended [43]. An appropriate postoperative pathway, using telemedicine in some cases, could be adopted. SICADS, in this regard, developed a Web App, “Con.Te SICADS” [44] with the aim to define an optimized health pathway for patients enrolled in an outpatient program. If any patient develops COVID-19 symptoms during the postoperative period, the specialist team must be immediately alerted, and all patients and professionals who had contact with the infected patient must be submitted to controls, even if vaccinated. Our goal is to protect patients by health professionals and to protect health staff by patients donning adequate PPE [41].

## 8 Discussion

This article is based on the review of recent studies about a new surgical organization during the COVID-19 pandemic and on the experience of Members of the Board of SICADS (Italian Society for Ambulatory ad Day Case Surgery). Our article is one of few concerning the resumption of the DS activity.

Peri- and postoperative COVID-19 complications significantly increase the risk of deaths and are considered a concern to defer or cancel nonurgent surgical services. Even in many high-income countries, the activities are limited to oncological and emergency surgery [11].

Surgery, however, should be maintained also during a pandemic, because it is an essential service of healthcare. Delays and cancellations may withhold patients from treatment that helps improve their quality of life.

The infection rate, indeed, could be underestimated [45], and the critical period may persist for many months. The risk for minor surgery is not negligible [46]; the risk related to COVID-19 adverse event, however, should be balanced against the deterioration of quality of life from delay or cancellation of the procedures.

Right now, following a long time of significant reduction of the minor procedures, surgical activity should be reopened, limiting the occurrence of infection. Otherwise, a high number of patients must be rescheduled in the future, and the healthcare system could collapse.

Waiting for a substantial change, over time, with the attainment of herd immunity by vaccines and specific medical care, full attention to limit any risk of infection must be taken.

DS can be considered at low risk due to its short duration, low complexity, and kind of anesthetic management, but needs a very strategic organization. This happened every time, more now during the COVID-19 pandemic. In fact, the best management for surgical elective patients must consider a short stay in hospital before, during, and after surgery. The question is how to safely organize the pathway. The 70% approximately of all kinds of surgery could be treated under DS, but a re-engineering of health pathways and hospital facilities could help to manage most surgical patients reducing to stay in hospital. We need a new model to carry out the visit of prehospitalization, a video call or video chat might be the first step to select the clinical risk of the patient and organize the best way of prehospitalization. The postoperative period could be also converted to a contactless way, using telemedicine, web call, and web apps, able to provide details and adequate information [47,48]. A new period has started, and we should change our thinking for a new model of health.

The goal of this kind of surgery is the separation of pathways with respect to the long-stay and emergency surgery, and this allows a lower risk of contamination during admission. The shortness of the admission time also reduces the risk of contamination.

The preoperative pathway is very subdued. The limited number of the preoperative procedures avoids long commutes of patients inside the hospital.

The surgical rooms dedicated to not producing droplets patients should be separated from other day cases procedures. The choice of the kind of anesthesia should be pointed to the easier possible.

The decontamination procedures after surgery must be very strict. This extends the time of each procedure and necessarily reduces their number per day. Any specific measures to limit the biological risk of contamination must be adopted [49].

All these recommendations significantly increase the costs of management of the operating rooms, the wards, and the health workers, in violation of the main principle, under which outpatient surgery is a low-cost procedure [50,51,52].

Considering costs sustained in the pandemic period examined for SPD, lab tests, services, and algorithmic paths used, it is possible to conclude that costs sustained for secure and adequate surgical management and to guarantee healthcare workers are 1.32%, higher than the cost in standard conditions. Effectively in the operating room and in the surgical department, the usage of these devices is normal; it is correct to add only the cost of COVID-19 pharyngeal nose swabs and a greater need for equipment. It is possible to guarantee a safe surgical pathway by a good organization, with no consequences on patients’ and healthcare workers’ safety.

The request for low complexity surgical procedures is nevertheless very high from the population of patients, and they are not less important than others.

At present, the waiting lists of this kind of surgical patients have significantly increased, and in this phase of decrease of incidence of COVID-19 cases, the surgical activity of DS can be safely and effectively restarted. Any delay can conduct to further prolongation of the waiting lists, with additional damage for the patients.
